# Crosstalk between DNA methylation and hypoxia in acute myeloid leukaemia

**DOI:** 10.1186/s13148-023-01566-x

**Published:** 2023-09-13

**Authors:** Sam Humphries, Danielle R. Bond, Zacary P. Germon, Simon Keely, Anoop K. Enjeti, Matthew D. Dun, Heather J. Lee

**Affiliations:** 1 School of Biomedical Sciences and Pharmacy, The University of Newcastle, Callaghan, NSW 2308 Australia; 2 Precision Medicine Research Program, Hunter Medical Research Institute, New Lambton Heights, NSW 2305 Australia; 3 Immune Health Research Program, Hunter Medical Research Institute, New Lambton Heights, NSW 2305 Australia; 4 Department of Haematology, Calvary Mater Hospital, Waratah, NSW 2298 Australia; 5 New South Wales Health Pathology, John Hunter Hospital, New Lambton Heights, NSW 2305 Australia

**Keywords:** DNA methylation, Hypoxia, Acute myeloid leukaemia, Epigenetics, Reactive oxygen species, Treatment outcomes

## Abstract

**Background:**

Acute myeloid leukaemia (AML) is a deadly disease characterised by the uncontrolled proliferation of immature myeloid cells within the bone marrow. Altered regulation of DNA methylation is an important epigenetic driver of AML, where the hypoxic bone marrow microenvironment can help facilitate leukaemogenesis. Thus, interactions between epigenetic regulation and hypoxia signalling will have important implications for AML development and treatment.

**Main body:**

This review summarises the importance of DNA methylation and the hypoxic bone marrow microenvironment in the development, progression, and treatment of AML. Here, we focus on the role hypoxia plays on signalling and the subsequent regulation of DNA methylation. Hypoxia is likely to influence DNA methylation through altered metabolic pathways, transcriptional control of epigenetic regulators, and direct effects on the enzymatic activity of epigenetic modifiers. DNA methylation may also prevent activation of hypoxia-responsive genes, demonstrating bidirectional crosstalk between epigenetic regulation and the hypoxic microenvironment. Finally, we consider the clinical implications of these interactions, suggesting that reduced cell cycling within the hypoxic bone marrow may decrease the efficacy of hypomethylating agents.

**Conclusion:**

Hypoxia is likely to influence AML progression through complex interactions with DNA methylation, where the therapeutic efficacy of hypomethylating agents may be limited within the hypoxic bone marrow. To achieve optimal outcomes for AML patients, future studies should therefore consider co-treatments that can promote cycling of AML cells within the bone marrow or encourage their dissociation from the bone marrow.

## Background

Characterised by the uncontrolled proliferation and diminished differentiation of immature myeloid cells in the bone marrow (BM), acute myeloid leukaemia (AML) is among the deadliest blood cancers [1]. Affecting ~ 5 in 100,000 individuals and carrying a dismal 5-year survival rate of ~ 25%, AML predominantly occurs in adults over the age of 60 [2, 3]. AML patients are typically treated with a standard combination of chemotherapies, such as daunorubicin and cytarabine [4]. While most patients respond well to these treatments and achieve remission, relapse often occurs within 3 years of diagnosis [5]. Unfortunately, many relapsed patients are typically non-responsive to further treatment, causing overall survival to be as low as 6 months from recurrence [6].

In comparison with most solid cancers, AML carries a relatively low mutational burden with an average of 13 mutations per patient [7, 8]. Among the most frequently mutated genes are several regulators of DNA methylation (*DNMT3A, TET2, IDH1/2*), showing that epigenetic dysregulation is an important driver of AML pathogenesis [9]. The BM microenvironment (BMME), in which AML develops, also influences disease progression through altered cell–cell interactions and extracellular factors [10, 11]. Since epigenetic mechanisms are known to respond to environmental stimuli [12], the crosstalk between the BMME and regulators of DNA methylation has clear relevance for AML. In this review, we consider how the low oxygen availability in the BMME may influence DNA methylation in AML. First, the importance of DNA methylation in AML development and treatment is described. Second, the effects of hypoxia on AML cells are summarised. Finally, we discuss likely modes of crosstalk between DNA methylation and hypoxia signalling, as well as implications for AML treatment.

## DNA methylation and AML

### Regulation of DNA methylation

DNA methylation is a critical component of the epigenome, with indispensable roles in regulating gene expression during development [13]. Methylation of the 5’ carbon in cytosine residues (5’-methylcytosine, 5mC) occurs in CpG dinucleotides in mammalian genomes. While majority of CpGs in the genome are methylated, short CpG-rich sequences termed CpG islands (CGIs) are generally hypomethylated. Most CGIs are located in gene promoters, and methylation of these loci can lead to transcriptional repression [14].

Regulation of DNA methylation is achieved through the combined actions of several enzyme families. De novo DNA methyltransferase (DNMT) enzymes, DNMT3A and DNMT3B, establish new DNA methylation marks within the genome [15, 16], while DNMT1 ensures that methylation profiles are stably inherited during cell division. In contrast, ten–eleven translocase (TET) enzymes initiate active DNA demethylation by oxidising 5mC to 5’-hydroxymethylcytosine (5hmC), and other modified bases that are then excised and replaced by DNA repair enzymes [17, 18]. Importantly, TET activity depends on several co-factors including oxygen and α-ketoglutarate (α-KG), which is produced by isocitrate dehydrogenase (IDH) enzymes [19, 20]. In AML, mutations have been detected in genes encoding DNMT3A, TET2, and IDH enzymes. These mutations disrupt DNA methylation patterns leading to dysregulated gene expression and altered differentiation (as outlined below).

### Dysregulation of DNA methylation in AML

DNMT3A, TET2, and IDH enzymes are all required for appropriate haematopoiesis [20–24], with loss of enzyme activity causing proliferation of immature cells, impaired differentiation, and lineage skewing [25–29]. Mutations in these epigenetic regulators are insufficient to trigger overt leukaemia, and are detected in healthy individuals [30, 31], clonal haematopoiesis [32], and pre-leukaemic myelodysplasias [33–36], with the frequency of mutations increasing with age [37]. Upon acquisition of an AML driver mutation (e.g. *FLT3*, *NPM1*), the expanded progenitor population transforms to leukaemia [9, 38]. Thus, mutations in epigenetic regulators reshape the genetic landscape that is permissive for AML development.

**Fig. 1:**
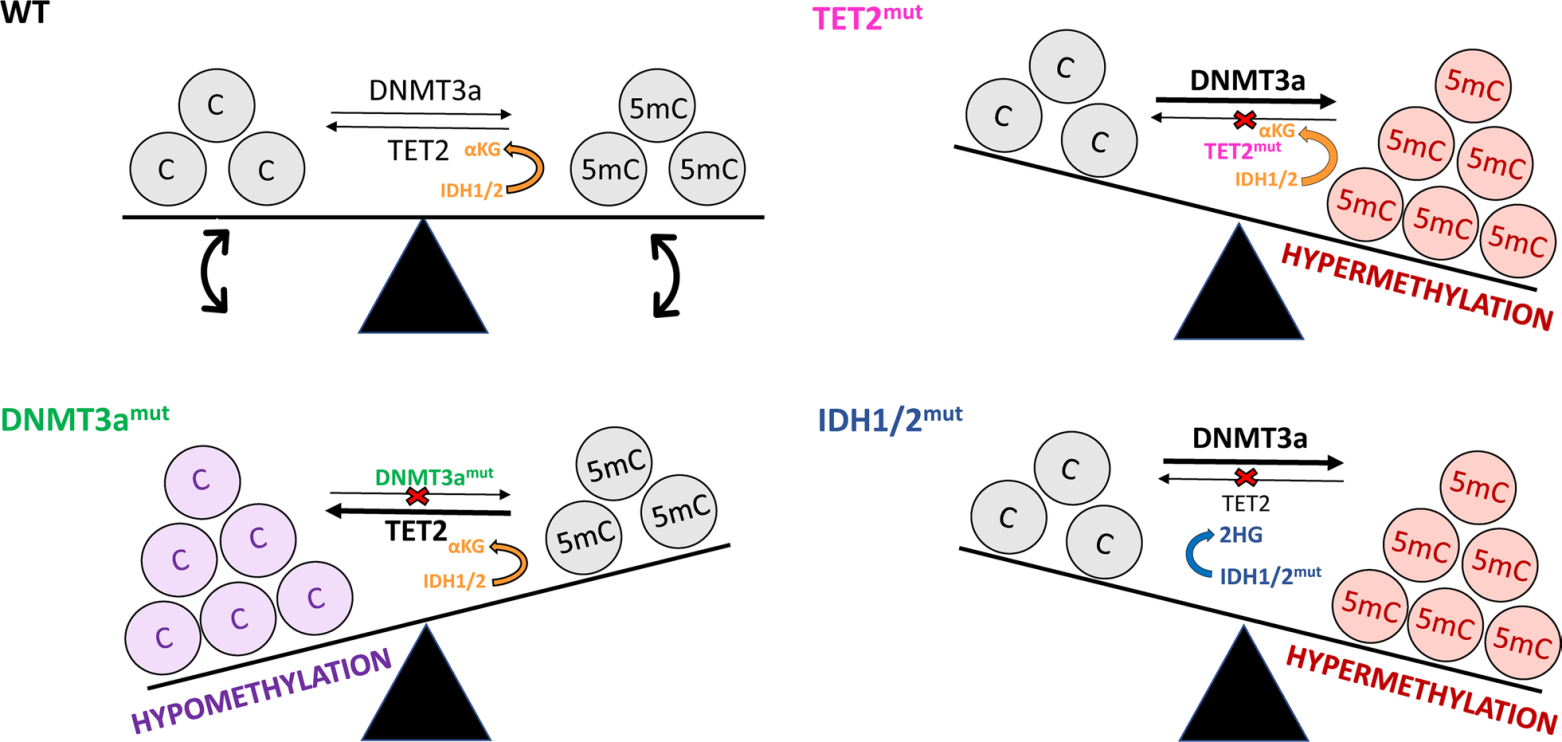
Common mutations in epigenetic regulators and their influence on DNA methylation in AML. In wild-type cells (black; top, left), the equilibrium between methylated (5mC) and unmodified (C) cytosines is governed by balanced DNMT3A and TET2 activity. IDH1/2 activity produces α-KG (orange), which is required for TET function. Loss of function *DNMT3A* mutations (green; bottom, left) lead to impaired DNMT3A activity and hypomethylation (purple). Conversely, loss of function *TET2* (pink; top, right) or *IDH1/2* (blue; bottom right) mutations result in hypermethylation (red). Mutations in *IDH1/2* lead to the production of the oncometabolite 2-HG, which acts as a competitive inhibitor for TET2, resulting in decreased TET2 activity and hypermethylation (blue; bottom, right)

*DNMT3A* mutations are observed in 12–22% of AML patients and are associated with reduced survival [39–42]. Missense mutations of arginine 882 (R882) are the most common abnormality [43, 44], causing reduced enzyme activity [45, 46] and altered patterns of DNA methylation [47]. While the global level of DNA methylation is not markedly affected [42], hypomethylation has been observed at many loci and in many genomic contexts [45, 48] (Fig. 1). The transcriptional consequences of these changes have been challenging to discern, since hypomethylated genes are not always upregulated in *DNMT3A*-mutant AML [42, 45, 47, 48]. Recently, single-cell analysis has been applied to resolve this issue. In clonal haematopoiesis patients with mosaic *DNMT3A* mutations, loss of DNA methylation at MYC binding sites in *DNMT3A*-mutant cells was accompanied by increased expression of MYC target genes [49]. Thus, changes in DNA methylation caused by *DNMT3A* mutations perturb key transcriptional programmes critical for myeloid differentiation.

*TET2* is mutated in ~ 20% of AML patients [38, 48, 50, 51] and is associated with unfavourable outcomes [38, 50, 52, 53]. Frameshift and nonsense mutations are spread across the whole *TET2* coding sequence, and missense mutations occur in two conserved domains that are important for enzyme function [38, 50]. Mutations in *TET2* prevent the conversion of 5mC to 5hmC [53, 54] and result in a hypermethylated phenotype that can encourage myeloid proliferation [55, 56] (Fig. 1). For example, in patients with clonal haematopoiesis and clonal cytopenia, *TET2* mutations were associated with increased methylation of enhancers linked to myeloid differentiation [57].

Mutations in *IDH1* or *IDH2* are found in ~ 10% of AML patients, with missense mutations common at IDH1 R132 and IDH2 R140 or R172 residues [58, 59]. IDH enzymes support the catalytic activity of TET and other oxoglutarate enzymes by producing their essential co-factor, α-KG [60]. However, mutant IDH enzymes produce an oncometabolite, 2-hydroxyglutarate (2-HG), which competes with α-KG [61–63]. In turn, TET activity is reduced by 2-HG, leading to a global reduction in 5hmC levels and subsequent increases in 5mC [53] (Fig. 1). Given that *IDH* mutations result in loss of TET function, they seldom co-occur with *TET2* mutations and are associated with hypermethylation of MYC target sites, as seen in *TET2*-mutant cells [20, 64]. While there are many similarities between *TET2* and *IDH*-mutant AMLs, it is important to note that TETs are not the only enzymes dependent on α-KG. Other members of the α-KG-dependent dioxygenase family include: an N^6^-methyladenosine RNA demethylase (e.g. FTO) [65], histone demethylases (e.g. KDM7A/2B/5C) [60], and prolyl hydroxylase domain (PHD) enzymes, which are negative regulators of hypoxia-inducible factors (HIFs) [66]. Thus, *IDH* mutations may impact multiple aspects of epigenetic regulation, as well as responses to hypoxia.

Intriguingly, common mutations in AML have contrary effects on DNA methylation: *DNMT3A* mutations are associated with hypomethylation, while *TET2* and *IDH1/2* mutations lead to increases in DNA methylation (Fig. 1). These mutations can also co-occur, with around 40% of *DNMT3A*-mutant cases carrying a mutation in either *TET2*, *IDH1* or *IDH2* [48]. This suggests that AML is not driven by specific patterns of DNA methylation, but rather by disruption of the epigenetic equilibrium within blast cells. As such, DNA methylation can be dysregulated and clinically relevant in AML, even when the mutations described above are absent.

One interesting source of dysregulated DNA methylation in AML is the high levels of reactive oxygen species (ROS) observed in patients [67–69]. While ROS and oxidative stress can deplete the DNMT co-factor S-adenosyl methionine (SAM) resulting in reduced enzyme activity [70, 71], ROS can also act directly on DNA to convert 5mC to 5hmC [71]. If production remains unregulated, ROS can convert guanine bases to 8-hydroxydeoxyguanosine (8-OHdG), inhibiting the maintenance of methylation at nearby cytosines [72, 73]. Oxidative DNA damage also influences the formation of epigenetic complexes including DNMT1, DNMT3B, and polycomb repressive complex 4, as well as promote tighter binding of DNMT1 to chromatin, trapping the enzyme and causing transcriptional repression [74].

Several studies have profiled DNA methylation in large patient cohorts revealing epigenetic differences between AML subtypes [75, 76]. New molecular subtypes with differences in patient survival were identified on the basis of DNA methylation alone [76]. High levels of 5hmC can also predict inferior survival [53], suggesting that active remodelling of the methylome may promote AML growth. More recent work has used computational approaches to estimate intra-tumoural DNA methylation heterogeneity in patient samples [77, 78]. These analyses have demonstrated that high DNA methylation heterogeneity is associated with specific genetic abnormalities (e.g. *IDH1/2* and *CEBPA* mutations) and reduced time to relapse. Information from 26 loci was sufficient to divide patients into low- and high-risk groups with significantly different relapse-free survival [77]. Dysregulated DNA methylation was associated with variable expression of neighbouring genes, suggesting that epigenetic heterogeneity allows diversification of transcriptional states within a tumour [77]. This may in turn promote AML progression by increasing the collective fitness of a population of cancer cells.

### AML therapies targeting DNA methylation

Given the important role of DNA methylation in the initiation and progression of AML, it is not surprising that therapeutics targeting DNA methylation are being used to treat AML patients.

#### Hypomethylating agents (HMAs)

DNA hypomethylating agents (HMAs) are used as alternatives to standard chemotherapies for older AML patients, due to their low toxicity. Decitabine (DAC, 2’-deoxy-5-azacytidine) and azacytidine (AZA, 5-azacytidine) are two HMAs approved for the treatment of AML and a pre-leukaemic dysplasia known as myelodysplastic syndrome (MDS). Early clinical trials showed that higher-risk MDS patients treated with AZA had significantly increased overall survival (OS) compared to the conventional care group (24.5 vs. 15 months) [79]. Similar results have been observed for DAC, and in various other patient groups [80–85].

Despite these benefits, the use of HMAs is limited by variable patient responses, with only 20–30% of patients benefitting from therapy [86]. Many studies have investigated genetic, epigenetic, and other determinants of patient response. Some have noted improved responses in patients with *DNMT3A* [87], *TET2* [88] or *IDH1/2* [89] mutations, while others have yielded contrary results [90]. Studies have also suggested that DNA methylation levels before treatment may be a better predictor of HMA response rather than changes induced by therapy [91]. Changes to the BMME may also influence HMA response, as indicated in a study of AZA treatment in MDS patients. Non-responding patients were found to have higher proportions of quiescent progenitor cells in the BM. These cells expressed high levels of integrin alpha 5, a cell-surface protein important for cell–extracellular matrix adhesion within the BMME [92]. Thus, interactions between malignant blasts and the BMME may influence HMA efficacy.

DAC and AZA are cytidine analogues that are incorporated into DNA during replication [93–96]. This leads to degradation of DNMT enzymes, loss of DNA methylation, decreased growth, and increased immunogenicity in AML cells [96–102]. HMA-induced promoter demethylation has been associated with re-expression of tumour suppressor genes; however, many studies also show wide-spread increases in gene expression that are independent of promoter demethylation [96, 99, 102, 103]. This suggests that not all transcriptional changes induced by HMA treatment are dependent on methylation changes at *cis*-regulatory elements. DAC and AZA can also trigger a ‘viral mimicry’ response by upregulating the expression of endogenous retroviral (ERV) elements scattered across the genome. These transcripts are then recognised by the viral defence pathway, promoting apoptosis via an interferon response [104–106]. AZA-responsive patients have also shown a greater upregulation of many transposable elements compared to non-responding patients, demonstrating the clinical relevance of viral mimicry [107].

#### IDH1/2 therapies

Therapies have recently been developed to target mutant IDH enzymes. Ivosidenib (AG-120) and enasidenib (AG-221) are now approved for treatment of *IDH1*- and *IDH2*-mutant cancers, respectively. By reducing 2-HG production, IDH inhibitors can trigger epigenetic reprogramming and restoration of myeloid differentiation in AML [108, 109]. However, these agents are currently only used in relapsed or refractory (r/r) AML cases, where standard treatments are no longer beneficial.

In patients with advanced *IDH1*-mutant AML, ivosidenib induced remission in 30.4% of patients, with an associated median OS of 14.5 months [110]. Similar benefits were observed in newly diagnosed *IDH1*-mutant AML patients ineligible for standard chemotherapy [111], and in clinical trials of enasidenib treatment in *IDH2*-mutant AML [112, 113]. Despite these promising results, patient responses remain variable, and relapse is common. As such, further studies are required to improve the clinical utility of IDH inhibitors.

#### Combination therapies

To sustain long-term treatment responses and improve patient survival, combinatorial treatment strategies are being designed to enhance the efficacy of epigenetic therapies.

IDH inhibitors are currently being tested in combination with HMAs to treat IDH-mutant AML patients. By reducing DNA methylation through DNMT inhibition, and simultaneously blocking 2-HG production to restore TET2 activity, this combination strategy may improve efficacy in patients. In a phase IB trial, newly diagnosed IDH1-mutant AML patients treated with ivosidenib and AZA showed deep and durable treatment responses (overall response rate (ORR) 78.3%; complete remission (CR) 60.9%; and 12-month survival estimate 82%) [114]. Promising results have also been obtained from a recent phase III clinical trial [115], studies combining enasidenib with AZA [116, 117], and pre-clinical studies of newly developed IDH inhibitors [118].

Venetoclax is a selective BCL-2 inhibitor that is widely used to promote apoptosis in haematological malignancies [119]. The benefits of combining venetoclax and HMA treatment have been shown in several studies of AML, including treatment-naïve and r/r cases [120–123]. For example, one study reported superior responses in r/r patients (64% ORR vs. 19% AZA alone), as well as patients with *IDH1/2* or *TP53* mutations (67% ORR) [120]. Combined HMA and venetoclax therapy has also enhanced survival in elderly treatment-naïve AML patients [123, 124], and improved outcomes in patients with MDS [125, 126], as well as those undergoing stem cell transplantation [127, 128].

E-selectin is an endothelial cell adhesion molecule typically found in the BM, which regulates haematopoietic stem cell (HSC) self-renewal, homing and engraftment potential [129]. In AML, leukaemic blasts can bind to E-selectin on endothelial cells, holding them within the BM to avoid the effects of certain chemotherapies. Currently, an E-selectin inhibitor known as uproleselan (GMI-1271) is in phase III clinical trials in combination with chemotherapy for r/r AML [130]. Uproleselan has been shown to release quiescent AML blasts into the cell cycle, blocking pro-survival pathways, and reducing the retention of blasts in the BM [129]. In turn, uproleselan may also enhance the efficacy of other therapies, like HMAs, that are particularly dependent on replication. Encouragingly, the combination of uproleselan with venetoclax and HMAs has demonstrated improved survival in pre-clinical models of AML [131], and in AML patients [132]. In a phase I clinical trial of elderly or treatment-naïve AML patients, 75% of patients achieved remission [132]. Overall, E-selectin inhibition is a promising therapeutic avenue that highlights the need to target AML cells in the context of the BM.

## Hypoxia and AML

### Hypoxia signalling

While we know that AML develops within the BM, the direct role of the microenvironment on leukemogenesis is only beginning to be elucidated. The BM contains two distinct hypoxic niches with differing capillary types: the endosteal region with thick-walled, low-permeable vessels that enforce low oxygen tensions (1% O_2_), and the more oxygen tense sinusoidal-vascular region (5% O_2_) that contains more fenestrated, permeable capillaries [133, 134].

For cells to sense, coordinate, and adapt to these low oxygen conditions, oxygen-sensing transcription factors known as hypoxia-inducible factors (HIFs) are required to activate hypoxia-responsive genes. In low oxygen conditions, oxygen-sensitive alpha (α) subunits (HIF1α, HIF2α, HIF3α) and constitutively expressed beta (β) subunits (HIF1β, HIF-2β) dimerise to form a HIF complex. These HIF complexes can then translocate into the nucleus, where they bind to hypoxia-responsive elements (HREs) in promoter or enhancer regions of hypoxia-responsive genes [135, 136]. HIF-induced transcription of these genes can modulate oxygen consumption (e.g. pyruvate dehydrogenase kinase 1—*PDK1*) [137], erythrocyte production (e.g. erythropoietin—*EPO*) [138], angiogenesis (e.g. vascular endothelial growth factor—*VEGF*) [139], mitochondrial metabolism (e.g. *IDH1,* cytochrome c oxidase subunit 4—*COX4*) [140, 141], and cellular quiescence (e.g. early growth response 1—*EGR1,* signal transducer and activator of transcription 5—*STAT5*) [142, 143]. Conversely, in regions with higher oxygen tension, HIFα subunits are modified by prolyl hydroxylase domains, von Hippel–Lindau proteins, or factor inhibiting HIF1 enzymes to trigger degradation or inhibition of HIFα complexes [144–146].

Hypoxia also triggers metabolic reprogramming. While oxidative phosphorylation (OXPHOS) is the predominant source of energy for cells in oxygen-rich tissues, anaerobic glycolysis is used in hypoxic regions. HIF1α transcription factors upregulate the expression of glucose transporter 1 (GLUT1 encoded by *SLC2A1*) [147] and lactate dehydrogenase A (*LDHA*) [148] to increase glucose intake and the downstream conversion of pyruvate to lactate, respectively. HIF1α further supports anaerobic glycolysis by upregulating *PDK1* expression, which prevents mitochondrial respiration and OXPHOS by inhibiting pyruvate dehydrogenase (PDH) [149]. Compared to OXPHOS, the energy generated by anaerobic glycolysis is very low (~ 2 ATP molecules vs. ~ 32 molecules, respectively) [150], meaning that suppressed cell growth is crucial for homeostasis in hypoxic conditions. OXPHOS also has the ability to generate ROS in the form of superoxide radicals (O_2_^−^) whereby the reduction of oxygen produces electrons that can leak from complexes in the electron transport chain. Superoxides are then converted to hydrogen peroxide (and O_2_) by mitochondrial superoxide dismutase’s (SODs) [151, 152].

Interestingly, ROS production within HPSCs is related to their proliferative state. Quiescent HSCs with low-cycling rates are associated with low levels of ROS production, while HSPCs with short-term repopulating capacity and increased cycling have higher ROS production [153, 154]. Thus, HSCs can use the endosteal niche to enter a quiescent state that protects them from oxidative DNA damage [154].

### Effects of hypoxia in AML

The effects of hypoxia on AML cells and patients are complex, with in vitro and in vivo analyses yielding contradictory results [155–163]. Some evidence suggests that AML cells are capable of proliferating rapidly within the hypoxic BM, while other data suggest that they can avoid the effects of chemotherapy by entering quiescence within hypoxic microenvironments.

Several studies have demonstrated that increased *HIF1A* expression in AML is associated with upregulation of genes such as *VEGF, GLUT1*, and heme oxygenase-1 (*HO-1*); encouraging disease progression through increased angiogenic, metabolic, and apoptotic processes, respectively [164–167]. These effects are reversed with HIF1α inhibition, suppressing the growth of AML cell lines, and encouraging various apoptotic pathways [166]. The glycolytic switch induced by hypoxia can also promote AML cell growth, viability, and survival [160, 168–172]. A distinct glycolytic profile is observed in AML patients, with high levels of glycolytic metabolites predicting poor survival [173]. Furthermore, markers of anaerobic glycolysis are increased in patients who do not achieve remission [165]. These studies suggest that AML cell growth is encouraged within the hypoxic BM, and accordingly, pre-clinical studies show that AML blasts can outcompete healthy myeloid cells [158]. Importantly, this over-proliferation of AML cells generates an increasingly hypoxic environment that forces healthy HSCs to enter quiescence [159].

In contrast to the studies described above, hypoxia can also suppress AML cell growth in certain circumstances. Most in vitro studies of AML cell lines have reported increased *HIF1A* expression, together with reduced cell growth, in the context of low oxygen environments [155–157]. HIF1α can encourage entry of AML blasts into the G_0_/G_1_ phase of the cell cycle, while upregulating an S-phase inhibitory protein, known as p27 [157], which can enhance resistance to replication-dependent drugs such as cytarabine (Ara-C) [174, 175]. Similarly, HIF1α-induced GLUT1 activity has been associated with poor therapeutic response in AML [165, 176]. Together, these studies suggest that leukaemic stem cells (LSCs) may localise to the hypoxic BM environment following chemotherapy [177], where a glycolytic shift and resulting quiescence can protect them from treatment. To circumvent this possibility, pre-clinical studies are exploring the benefits of therapeutically targeting hypoxia-induced signalling and metabolic reprogramming in haematological tumours [69, 178, 179].

A role for HIF2α in AML is also becoming more evident, where blast cells in the BM of leukaemic mice exhibit higher *HIF2A* expression compared to healthy mice. AML cell lines support this, demonstrating that *HIF2A* deletion can decrease cell proliferation and prolong survival in xenograft experiments [180]. Knock-out in primary AML cells also lowered the engraftment and survival capacity of those cells [181].

In summary, the capacity of AML cells to survive and thrive in hypoxic environments is a contributor to poor prognosis [159, 182]. Within the BM, AML blasts can proliferate rapidly during disease progression, while LSCs lie quiescent to avoid chemotherapy. Optimised AML treatments will depend on a thorough understanding of hypoxia signalling in AML cells, as well as strategies to prevent the rapid adaptation of AML cells to changes in their environment.

## Crosstalk between hypoxia and DNA methylation

As described above, both epigenetic regulation and the hypoxic BM play important roles in AML development and progression. Here, we explore the interactions between hypoxia and DNA methylation in AML, as well as any implications for therapeutic efficacy.

### Hypoxia and TETs

**Fig. 2:**
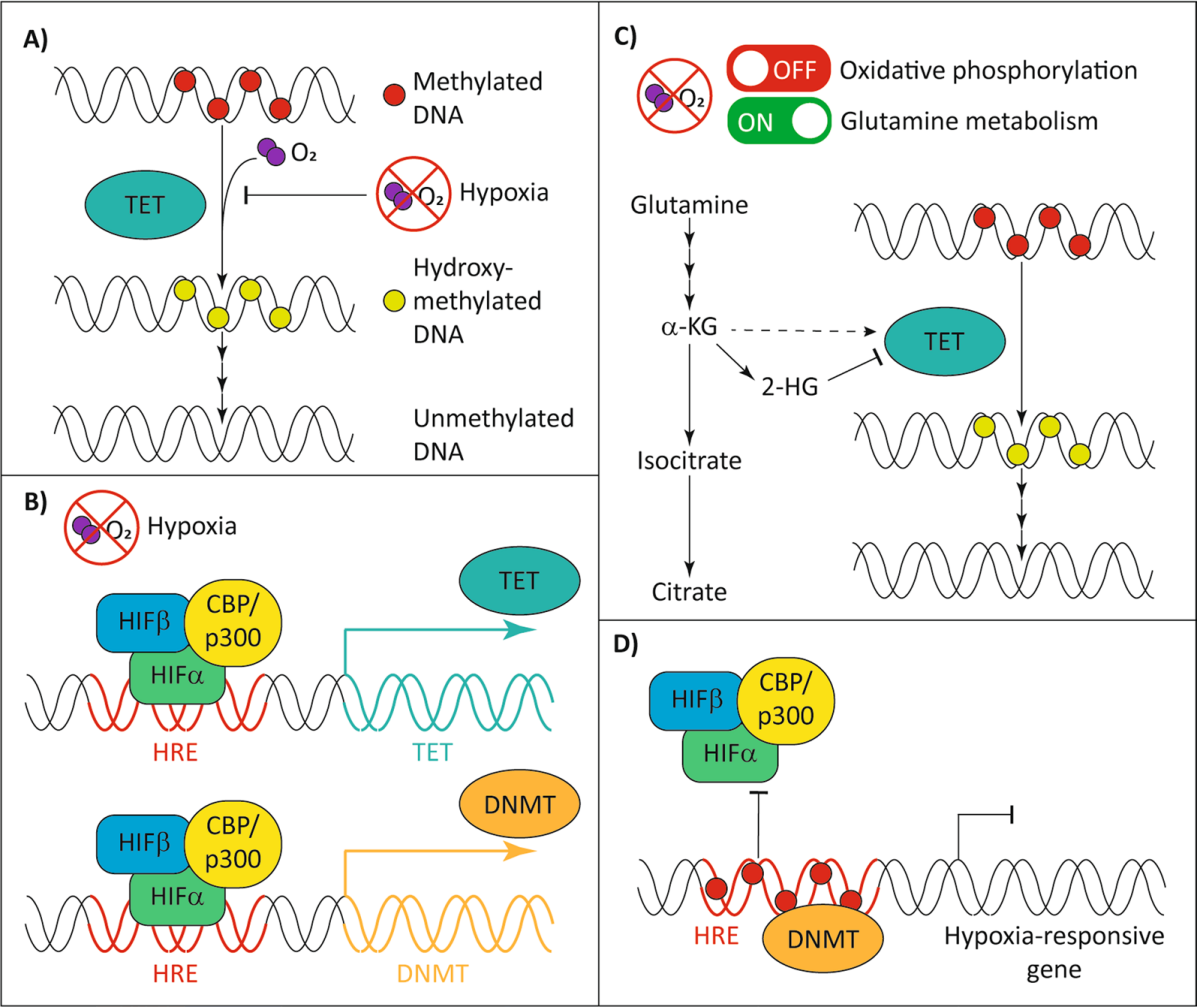
Complex interactions between hypoxia and DNA methylation. Hypoxia may influence DNA methylation via direct effects on TET activity, altered transcription, or metabolic reprogramming. DNA methylation may also influence hypoxia responses. **A** Oxygen is an essential co-factor for TET enzymes, and hypoxia reduces TET-mediated DNA hydroxymethylation in some cancers. **B** In certain cell types, hypoxia-inducible factors (HIFα, HIFβ) bind to hypoxia-responsive elements (HREs) in *TET* and *DNMT* promoters to induce their expression. **C** In hypoxia, cancer cells can induce a metabolic switch from oxidative phosphorylation to glutamine metabolism. In *IDH* wild-type cells, upregulated glutamine metabolism has been associated with production of 2-HG which can inhibit TET enzymes. **D** DNA methylation can prevent the binding of HIF complexes to HREs, altering transcriptional responses induced by hypoxia

During active demethylation, TET enzymes require oxygen to convert 5mC to 5hmC (Fig. 2a), implying that hypoxia may limit TET activity to create a hypermethylated state. In a study conducted by Thienpont et al., 5hmC levels were significantly decreased across 11 cancer cell lines in hypoxia (0.5% O_2_), with *TET* expression showing a positive correlation with 5hmC levels [54]. Further investigations using MCF7 breast cancer cells revealed that reductions of 5hmC in hypoxia were accompanied by increases in 5mC levels, with changes especially pronounced at gene enhancers, promoters, and actively transcribed regions.

HIF signalling can also influence TET expression and activity depending on the cancer type (Fig. 2b). For example, in the hypoxic microenvironment of metastatic melanoma and glioblastoma, knockdown of HIF1α was associated with increased *TET2* expression and 5hmC levels [183], while in neuroblastoma [184, 185] and hepatocellular carcinoma [186] studies, *TET* expression and 5hmC levels were increased in hypoxia. The above study by Thienpont also performed ChIP-seq to demonstrate HIF binding within *TET* promoter regions [54].

Until recently, the relationship between hypoxia and TET activity in AML had not been explored. In 2021, an analysis of KG-1 AML cells exposed to hypoxia (1–3% O_2_) demonstrated a positive correlation between *HIF1A* and *TET2* expression [170]. Enhanced *TET2* transcription was also associated with increased binding of HIF1α to the *TET2* promoter, which subsequently increased 5hmC and decreased 5mC. As such, HIF1α-induced TET expression may override any reduction in TET activity caused by low oxygen availability in the context of AML.

TET3 may also be regulated by hypoxia in haematological malignancies. In a chronic myeloid leukaemia cell line (K562), the enhancer for *TET3* was identified as a strong target for HIF1α binding, with *TET3* expressed at higher levels than *TET1* and *TET2* in hypoxia [187]. Deletion of HIF1α binding sites in the *TET3* enhancer resulted in decreased *TET3* expression that reduced cell viability and impaired erythroid differentiation.

Overall, these studies demonstrate that the effects of hypoxia on *TET* expression and activity may be tissue dependent. In some cases, like leukaemia, hypoxia can enhance *TET2* or *TET3* expression to induce hypomethylation, while in others like breast cancer, it can reduce TET activity and promote hypermethylation across the genome.

### Hypoxia and IDH1/2

Hypoxia can also influence the availability of the TET co-factor, α-KG. IDH enzymes, particularly IDH2, function in the mitochondria to convert isocitrate metabolites of the tricarboxylic acid (TCA) cycle into α-KG. However, when cells are deprived of oxygen, IDH metabolism and α-KG production are altered, influencing downstream TET activity and hence epigenetic regulation.

While hypoxia is well known to induce a glycolytic switch, some studies have found that hypoxia can also prompt a shift from OXPHOS to the glutamine-derived metabolic cycle (Fig. 2c). In *IDH* wild-type glioblastomas, hypoxia not only decreased TCA cycle activity, but also concomitantly increased glutamine-derived α-KG and the oncometabolite, 2-HG [188–190]. While α-KG can be produced by catabolism of glutamine, a non-reductive form of carboxylation can generate 2-HG without any *IDH* mutation [191]. Thus, hypoxia can mimic the effects of mutant IDH enzymes by generating 2-HG through alternate metabolic pathways.

In *IDH*-mutant cancers, the production of 2-HG can indirectly mimic a hypoxic state by promoting HIF1α activity [192]. PHDs are α-KG-dependent enzymes that typically ubiquitinate HIF1α in normoxic conditions. As a result, the production of 2-HG can reduce PHD activity, stabilising HIF1α to transcribe hypoxia-responsive genes. Thus, mutations in *IDH* have the potential to increase hypoxia signalling via loss of negative regulation [192, 193].

The production of 2-HG in primary *IDH*-mutant AML cells also inhibits the activity of cytochrome C oxidase (COX; Complex IV) enzymes that break down oxygen in the mitochondria for aerobic energy generation [194]. By decreasing the activity of Complex IV, oxygen consumption is reduced, such that the cell’s metabolic processes mimic a hypoxic state. This can create a glycolytic or glutaminergic metabolic shift that increases the anti-apoptotic effects of BCL-2 [195, 196], encouraging disease survival.

While studies in leukaemia are limited, a relationship between hypoxia and IDH enzymes is beginning to emerge. On the one side, hypoxia itself can cause a metabolic switch in *IDH* wild-type cells that indirectly produces 2-HG and inhibits α-KG-dependent enzymes, like TETs. On the other hand, production of 2-HG in cells with *IDH* mutations can promote a hypoxic-like state by either: promoting HIF signalling through PHD inhibition or decreasing OXPHOS via inhibition of Complex IV enzymes in the mitochondria.

### Hypoxia and DNMTs

Hypoxia also influences the expression of DNMT enzymes in cancers such as liver, prostate, and breast cancer. Increased activity of HIF1α in hypoxia is associated with enhanced binding to the *DNMT1* and *DNMT3B* promoters (Fig. 2b), promoting methylation and repression of tumour suppressor gene expression, such as protein sprouty homolog 2 (*SPRY2*) [197, 198]. Furthermore, DNMTs can act in a negative feedback loop to suppress *HIF* expression. In foetal lung fibroblasts, HIF2α*-*induced *DNMT1* expression was followed by methylation of the *HIF2A* promoter, which in turn dampened the hypoxic response (Fig. 2d) [199]. Renal cell carcinomas and glioblastoma cell lines support this finding, where reduced *DNMT3A* expression was associated with decreased *HIF2A* promoter methylation and increased *HIF2A* expression [200]. The ectopic expression of DNMT3A in hypoxia (1% O_2_) was also shown to impair cell proliferation and viability by reducing *HIF2A* mRNA expression and protein activity.

DNA methylation may also impact hypoxia responses by modulating HIF binding across the genome (Fig. 2d). In one study, *DNMT*-triple knockout MCF7 breast cancer cells demonstrated preferential binding of HIF1β to promoter and enhancer regions containing unmethylated HREs [201]. HIF1β ChIP-seq also demonstrated that methylation of these HRE sites reduced HIF1β binding 12.4-fold.

Overall, evidence suggests that there are complex relationships between DNMTs, DNA methylation, and hypoxia in cancer. HIF heterodimers have a higher affinity for unmethylated HRE motifs, implying that DNA methylation can limit hypoxia responses. Further, transcriptional changes induced by DNMT and HIF enzymes can be modulated through negative feedback. Therefore, a greater understanding of these interactions will be critical for optimal use of epigenetic therapies in AML.

### Effect of hypoxia on DNA methylation therapies in AML

Since hypoxia and DNA methylation both play important roles in AML, the bidirectional crosstalk described above is likely to have important clinical implications. For example, the hypoxic BMME could influence the efficacy of AML therapies targeting DNA methylation.

Given that HMAs are incorporated into DNA during replication, quiescent or low-cycling LSCs in the hypoxic BM niche may not respond to treatment. Consistent with this idea, MDS and AML patients who did not respond to AZA had a higher proportion of quiescent progenitor cells in the BM [87–92, 202, 203]. These LSCs could later resume cycling and promote relapse, suggesting that the long-term efficacy of HMAs depends upon uptake in cycling AML cells [127, 204–206]. One interesting pre-clinical study altered administration schedules to increase the proportion of cells exposed to HMA treatment during S-phase [206]. Continuous, twice-weekly administration of DAC was found to be more effective than 5 consecutive days of treatment followed by 3 weeks off therapy, suggesting that the treatment schedule used in current clinical practice may be sub-optimal.

HMA co-treatment schedules that increase AML cell cycling are also being considered. For example, E-selectin inhibitors may enhance HMA efficacy by forcing AML cells to move out of the hypoxic BM and resume cycling [207], encouraging the uptake of HMAs and a better treatment response. IDH1 inhibitors combined with AZA have also shown promise in treating AML by increasing the cycling of LSCs, allowing AZA to more effectively target these cells [208].

The key to improving treatment efficacy and long-term survival for AML patients may lie in combination therapies that target not only cancer cell proliferation, survival, or DNA methylation changes, but also their interactions with the hypoxic BM microenvironment. Such a treatment regime may aid in preventing or at least delaying relapse in AML.

## Conclusion

Hypoxia signalling within the BMME, and dysregulation of DNA methylation, both contribute to the development of AML. As outlined above, complex bidirectional interactions between hypoxia and DNA methylation are likely to influence AML cell proliferation, with important clinical implications. Specifically, epigenetic therapies such as HMAs may have limited efficacy in the hypoxic BM due to reduced cell cycling in this microenvironment. Consideration of the interactions between the epigenome and the microenvironment in AML will lead to improved outcomes for patients.

## Data Availability

Not applicable.

## Abbreviations

BM: Bone Marrow
AML: Acute Myeloid Leukaemia
BMME: Bone Marrow Microenvironment
5mC: 5’-Methylcytosine
CGIs: CpG Islands
DNMT: DNA Methyltransferases
TET: Ten–Eleven Translocases
5hmC: 5’-Hydroxymethylcytosine
α-KG: α-Ketoglutarate
IDH: Isocitrate Dehydrogenases
2-HG: 2-Hydroxyglutarate
PHD: Prolyl Hydroxylase Domains
HIF: Hypoxia-Inducible Factors
ROS: Reactive Oxygen Species
HMAs: Hypomethylating Agents
DAC: Decitabine; 2’-deoxy-5-azacytidine
AZA: Azacytidine; 5-azacytidine
MDS: Myelodysplastic Syndrome
OS: Overall Survival
ERV: Endogenous Retroviral
ORR: Overall Response Rates
CR: Complete Remission
r/r: Relapsed or Refractory
HSCs: Haematopoietic Stem Cells
HRE: Hypoxia-Responsive Element
VHL: Von Hippel–Lindau
FIH-1: Factor-Inhibiting HIF1
Ara-C: Cytarabine
OXPHOS: Oxidative Phosphorylation
PDK: Pyruvate Dehydrogenase Kinase
PDH: Pyruvate Dehydrogenase
ATP: Adenosine-5’-Triphosphate
SOD: Superoxide Dismutase
HSPCs: Haematopoietic Stem and Progenitor Cells
LSC: Leukaemic Stem Cell
TCA: Tricarboxylic Acid
COX: Cytochrome C Oxidase
ETC: Electron Transport Chain
